# Investigating the Radiobiological Response to Peptide Receptor Radionuclide Therapy Using Patient-Derived Meningioma Spheroids

**DOI:** 10.3390/cancers16142515

**Published:** 2024-07-11

**Authors:** Thom G. A. Reuvers, Vivian Grandia, Renata M. C. Brandt, Majd Arab, Sybren L. N. Maas, Eelke M. Bos, Julie Nonnekens

**Affiliations:** 1 Department of Molecular Genetics, Erasmus MC Cancer Institute, Erasmus University Medical Center, 3015 GD Rotterdam, The Netherlands; 2 Department of Radiology and Nuclear Medicine, Erasmus MC Cancer Institute, Erasmus University Medical Center, 3015 GD Rotterdam, The Netherlands; 3 Department of Neurosurgery, Erasmus MC Cancer Institute, Erasmus University Medical Center, 3015 GD Rotterdam, The Netherlandse.bos@erasmusmc.nl (E.M.B.); 4 Department of Pathology, Leiden University Medical Center, 2333 ZA Leiden, The Netherlands; 5 Department of Pathology, Erasmus MC Cancer Institute, University Medical Center Rotterdam, 3015 GD Rotterdam, The Netherlands

**Keywords:** meningioma, peptide receptor radionuclide therapy, external beam radiotherapy, radiobiology, patient-derived in vitro models

## Abstract

**Simple Summary:**

Peptide receptor radionuclide therapy (PRRT) is a potential new treatment option for patients with meningiomas, the most common form of adult intracranial brain tumors. During PRRT, targeted radiation is delivered to the tumor through a radiolabeled peptide binding a receptor commonly overexpressed in meningioma cells. Unfortunately, preclinical research into therapy effects is limited due to the lack of appropriate in vitro models. Here, we aimed to develop an easy-to-use, scaffold-free patient-derived 3D culture model to support this research.

**Abstract:**

Peptide receptor radionuclide therapy (PRRT) using ^177^Lu-DOTA-TATE has recently been evaluated for the treatment of meningioma patients. However, current knowledge of the underlying radiation biology is limited, in part due to the lack of appropriate in vitro models. Here, we demonstrate proof-of-concept of a meningioma patient-derived 3D culture model to assess the short-term response to radiation therapies such as PRRT and external beam radiotherapy (EBRT). We established short-term cultures (1 week) for 16 meningiomas with high efficiency and yield. In general, meningioma spheroids retained characteristics of the parental tumor during the initial days of culturing. For a subset of tumors, clear changes towards a more aggressive phenotype were visible over time, indicating that the culture method induced dedifferentiation of meningioma cells. To assess PRRT efficacy, we demonstrated specific uptake of ^177^Lu-DOTA-TATE via somatostatin receptor subtype 2 (SSTR2), which was highly overexpressed in the majority of tumor samples. PRRT induced DNA damage which was detectable for an extended timeframe as compared to EBRT. Interestingly, levels of DNA damage in spheroids after PRRT correlated with SSTR2-expression levels of parental tumors. Our patient-derived meningioma culture model can be used to assess the short-term response to PRRT and EBRT in radiobiological studies. Further improvement of this model should pave the way towards the development of a relevant culture model for assessment of the long-term response to radiation and, potentially, individual patient responses to PRRT and EBRT.

## 1. Introduction

Meningiomas are the most common intracranial tumors in adults, most likely originating from arachnoid cap cells located in the meninges lining the brain and spinal cord [1]. For symptomatic tumors, which cause noticeable symptoms due to their location and size, surgical resection is the treatment of choice [2]. In contrast, for growing tumors, which are primarily identified by their increase in size over time, both surgical resection and radiotherapy are possible treatment options. The risk of post-operative tumor recurrence is dependent on the extent of resection and tumor aggressiveness [3]. External beam radiotherapy (EBRT) is used for recurrent WHO grade 1 meningiomas and as standard-of-care for more aggressive (WHO grade 2 and 3) meningiomas [4,5]. However, due to a lack of clinical evidence, there is still debate on the optimal use of radiotherapy in different patient groups [5], especially for meningiomas located close to eloquent areas such as the optic nerve, hippocampus, or pituitary gland. Unfortunately, preclinical data on meningioma (radio)biology to better understand the effects of ionizing radiation (IR) and improve therapy options are relatively limited [6].

Considering that meningiomas are located outside the blood–brain barrier and that high expression of somatostatin receptor subtype 2 (SSTR2) has been detected across all grades [7,8], peptide receptor radionuclide therapy (PRRT) employing [^177^Lu]Lu-[DOTA-Tyr^3^]octreotate (^177^Lu-DOTA-TATE) has recently been evaluated as a treatment option for meningiomas, mostly in a salvage therapy setting [9,10]. During PRRT, SSTR2 on the tumor cell surface is targeted by the somatostatin analog DOTA-TATE, after which the coupled radionuclide lutetium-177 locally induces DNA damage after radioactive decay [11]. This therapy is currently implemented in treatment schemes for patients with gastroenteropancreatic neuroendocrine tumors (GEP-NETs) [12]. At the moment, there is a lack of (pre)clinical data on the efficacy and safety of PRRT in meningiomas and other brain tumors. This is partly caused by the limited availability of culture models sufficiently mimicking meningioma biology [13]. Currently, models have been developed that range from immortalized cell lines to mouse models, such as patient-derived xenografts or genetically engineered mouse models, and primary 2D cell cultures [13,14]. Because of the radiobiological differences in 2D vs. 3D systems [15,16], as well as the influence of patient-specific characteristics on IR-therapy response in vivo [17], there is a clear need for patient-derived 3D models. These models should ideally retain the molecular and histological features (e.g., expression of meningioma markers such as SSTR2), proliferation rate, and cell type composition of the parental tumor. Recently, initial efforts have been made towards the development of these models, such as organoids [18,19]. However, further developments will be necessary to develop a reproducible patient-derived meningioma culturing system that can aid in radiobiological research, both for EBRT and PRRT. 

Here, we demonstrate the proof-of-concept for an easy-to-use 3D meningioma culture model based on tumor tissue-originated spheroids to use in EBRT and PRRT radiobiological research. By retaining cell–cell contact during tumor dissociation and spheroid preparation, the original tumor architecture was kept intact and the formation of spheroids was possible within a short time frame. Furthermore, we showed the feasibility of this system as a short-term patient-derived tumor model to evaluate the short-term DNA damage response induced by EBRT and PRRT. Further optimization of this model should pave the way to a system for evaluating the long-term response to targeted radionuclide therapy moieties such as PRRT in a highly relevant culture model that could be used clinically in a personalized therapy selection setting.

## 2. Materials and Methods

### 2.1. Patient Samples

All samples were retrieved as left-over material from meningioma patients undergoing surgical resection at the Erasmus University Medical Center (Erasmus MC), Rotterdam, The Netherlands. Informed consent was obtained from each patient. This study was approved by the local Medical Ethical Committee (protocol number MEC-2013-090). After resection, tissues were stored in cold transport medium (DMEM/F12 (Gibco, Bleiwsijk, The Netherlands) + 1% penicillin-streptomycin (PS; Sigma-Aldrich, Zwijnendrecht, The Netherlands)) and kept at 4 °C for a maximum of 2 h before transfer to the laboratory on ice and further processing. Tissue was coded anonymously so it was not traceable to corresponding patients by laboratory personnel. 

### 2.2. Isolation of Tissue Fragments

Tumor tissue was visually inspected and large blood clots, necrotic, or other non-tumor regions were removed using scalpels. Larger tumor pieces were cut into smaller pieces of 1–2 mm^3^, which were washed in cold 1× phosphate-buffered saline (PBS; Lonza, Geleen, The Netherlands). Multiple pieces from different regions of the tissue sample were fixed in formalin (J.T. Baker, VWR International, Amsterdam, The Netherlands) for 24 h at room temperature (RT) as baseline control samples. The remaining tissue pieces were enzymatically dissociated in DMEM/F12 + 1% PS containing 50 ng/mL Liberase TM (Sigma-Aldrich), supplemented with 0.9 U/mL Dispase II (Sigma-Aldrich) and 0.19 mg/mL DNAse I (Sigma-Aldrich) under orbital shaking (90 rpm) at 37 °C and 5% CO_2_. The suspension was visually examined every 30 min under a microscope and stopped when sufficient dissociated fragments and single cells were visible. The maximum dissociation time was 1 h to prevent excessive stressing of tissue. The suspension of dissociated fragments and single cells was collected in fresh DMEM/F12 + 1% PS and filtered through a 500 µm strainer (Pluriselect, Leipzig, Germany) to remove large tissue fragments. The filtrate was subsequently filtered through a 40 µm strainer, after which filtered tissue fragments with a size range of 40–500 µm were resuspended in DMEM/F12 + 1% PS by gentle pipetting. If a high amount of residual red blood cells (RBCs) was noted by a reddish color of the fragment pellet after centrifugation, fragments were additionally treated with homemade RBC lysis buffer (0.155 M NH_4_Cl, 0.1 mM EDTA and 10 mM KHCO_3_ in H_2_O) for 10 min at 4 °C. Fragments were washed in cold PBS three times by gentle centrifugation (200× *g*, 1 min) and resuspending, and microscopically inspected for intactness and general morphology afterward.

### 2.3. Culturing of Meningioma Spheroids

Obtained tumor fragments were resuspended in either growth factor-supplemented medium (DMEM/F12 + Neurobasal medium in a 1:1 ratio, supplemented with 1× N-2 supplement (Gibco, Bleiswijk, The Netherlands), 1× B-27 supplement (Gibco), 20 ng/mL epidermal growth factor (EGF), 20 ng/mL basic fibroblast growth factor (bFGF; Gibco), 2 mM L-glutamine (Stemcell Technologies, Vancouver, BC, Canada), and 1% penicillin-streptomycin (PS; Sigma-Aldrich) or DMEM/F12 supplemented with 10% fetal calf serum (FCS; Biowest, Nuaillé, France) and 1% PS. Fragments were divided equally over 6-well plates coated with a thin layer of 1% low-melting point agarose (Sigma) under continuous orbital shaking (30 rpm) at 37 °C and 5% CO_2_. The number of fragments per well was kept between 10 and 30 to prevent overcrowding. The medium was replaced every 3–4 days by collecting spheroids, gentle centrifugation, and resuspending in fresh medium. Total medium volume per well was 3 mL and spheroids were maintained under continuous orbital shaking.

### 2.4. Radiolabeling

^177^Lu-DOTA-TATE was used from left-over batches from standard labeling procedures of Lu-Mark (IDB Holland, Baarle-Nassau, The Netherlands) for patient treatment. The molar activity was 53 MBq/nmol, the radiochemical yield was >98% and the radiochemical purity was >95%.

### 2.5. EBRT and PRRT

For the application of EBRT, meningioma spheroids were irradiated in 6-well plates using an RS320 cabinet irradiator (X-Strahl, Walsall Wood, UK) at a dose rate of 1.6 Gy/min, to a cumulative absorbed dose of 2 Gy. For PRRT, different levels of activity of ^177^Lu-DOTA-TATE were added to a culture medium with meningioma spheroids in 6-well plates and incubated for 4 h under orbital shaking (50 rpm). Control spheroids were subjected to sham treatment. After 4 h, spheroids were collected, washed twice in PBS to remove unbound radioactivity, and used for further experimentation. To confirm specific uptake of PRRT, PRRT-treated spheroids were compared to spheroids co-incubated with 100× molar excess of non-radiolabeled DOTA-TATE (Auspep, Tullamarine, Australia) as a control condition that blocks the target receptor. For uptake experiments, spheroids were immediately processed. For the determination of 53BP1 foci, spheroids were collected immediately after treatment (PRRT and control, day 0), 2 h after treatment (EBRT, day 0), and at day 2 after treatment (all conditions).

### 2.6. Uptake Assays

After incubation with 0.1 MBq/mL or 1 MBq/mL ^177^Lu-DOTA-TATE and washing steps, spheroids were collected in 1 mL PBS and transferred to gamma counter tubes. Radioactivity was measured using a 1480 WIZARD automatic γ-counter (Perkin Elmer). PRRT-treated spheroids were compared to spheroids co-incubated with DOTA-TATE.

### 2.7. Hematoxylin and Eosin, Immunohistochemical and Immunofluorescent Stainings

For all stainings, parental tumor tissue fragments or cultured spheroids were fixed at indicated timepoints in 10% formalin (J.T. Baker) for 24 h at RT. Spheroids were counterstained with hematoxylin for visualization during sectioning, embedded in Histogel (Corning, Amsterdam, The Netherlands), and, subsequently, paraffin. Sections of 4 µm thickness were generated using a microtome. Sections were deparaffinized and rehydrated using standard protocols. Hematoxylin and eosin (H&E) stainings were performed as described previously [20]. For immunohistochemical (IHC) and immunofluorescent (IF) stainings, antigen retrieval was performed by boiling in 1× antigen retrieval buffer (pH6, DAKO, Agilent, Santa Clara, CA, USA) for 15 min. For IHC stainings, slides were incubated in 3% H_2_O_2_ in methanol for 20 min at RT to block endogenous peroxidase activity and blocked in 5% Protifar (Nutricia, Zoetermeer, The Netherlands) in 0.1% Triton X-100 in PBS. Sections were then incubated overnight with primary antibody in 1% Protifar in 0.1% Triton X-100 in PBS at 4 °C. The next day, slides were incubated in a secondary antibody for 1 h at RT. Visualization was conducted using the Liquid DAB+ substrate chromogen system (DAKO). Finally, slides were counterstained in hematoxylin and dehydrated before embedding using a PERTEX mounting medium (VWR). Visualization of H&E and IHC stainings was conducted using a BX-40 microscope (Olympus, Leiderokdorp, The Netherlands).

For IF stainings, samples were permeabilized using PBS + 0.5% Triton X-100 and blocked in 2% bovine serum albumin (BSA) in PBS + 0.1% Triton X-100. Primary antibody incubation was preformed in a blocking buffer for 90 min at RT in the dark, followed by secondary antibody incubation in a blocking buffer for 1 h. Sections were counterstained by incubation in 1 µg/mL DAPI (Thermo Fisher Scientific, Bleiswijk, The Netherlands) in PBS for 10 min before they were mounted using Vectashield (Vector Laboratories, Newark, CA, USA). Visualization of IF stainings was conducted on an LSM-700 confocal microscope (Zeiss, Oberkochen, Germany) using 40× and 63× oil immersion objectives.

### 2.8. Imaging of DNA Damage Repair Foci

For the determination of the number of 53BP1 foci per nucleus after EBRT (2 Gy) or PRRT (1 MBq/mL), Z-stacks were acquired at four randomly selected fields of view from sections from different spheroids per condition. The creation of maximum intensity Z-projections, nucleus segmentation, and determination of the number of 53BP1 foci per nucleus was conducted in FIJI using homemade macros. Nuclei were segmented based on the DAPI-channel using the StarDist plugin [21]. In each nucleus, the amount of 53BP1 foci was determined based on thresholding the Alexa Fluor 594 channel with a defined minimum and maximum size of foci. At least 130 cells were analyzed per condition, with a typical amount of 300–400 analyzed cells per condition.

### 2.9. Antibodies

For IF and IHC stainings, primary antibodies for SSTR2 (Abcam, Cambridge, UK; ab134152, 8.15 µg/mL), 53BP1 (Millipore, Amsterdam, The Netherlands; MAB3802, 1 µg/mL), and Ki-67 (DAKO M7240, clone MIB1, 0.48 µg/mL) were used. Secondary antibodies were donkey-anti-rabbit Alexa Fluor 488 (Invivogen, San Diego, CA, USA; A21206, 2 µg/mL), donkey-anti-mouse Alexa Fluor 594 (Invivogen A21203, 2 µg/mL), peroxidase-conjugated AffiniPure donkey-anti-rabbit IgG (Jackson ImmunoResearch, West Grove, PA, USA, 711-035-152, 8 µg/mL), and peroxidase-conjugated AffiniPure sheep-anti-mouse IgG (Jackson ImmunoResearch 515-035-003, 8 µg/mL).

### 2.10. Statistical Analyses

Statistical analysis was conducted in Graphpad Prism (version 9). For 53BP1 foci analyses, we used one-way ANOVA with Tukey’s test for multiple comparisons. All statistical analyses with *p*-value ≤ 0.05 were considered significant.

## 3. Results

### 3.1. Establishment of Meningioma Spheroid Cultures

**Figure 1:**
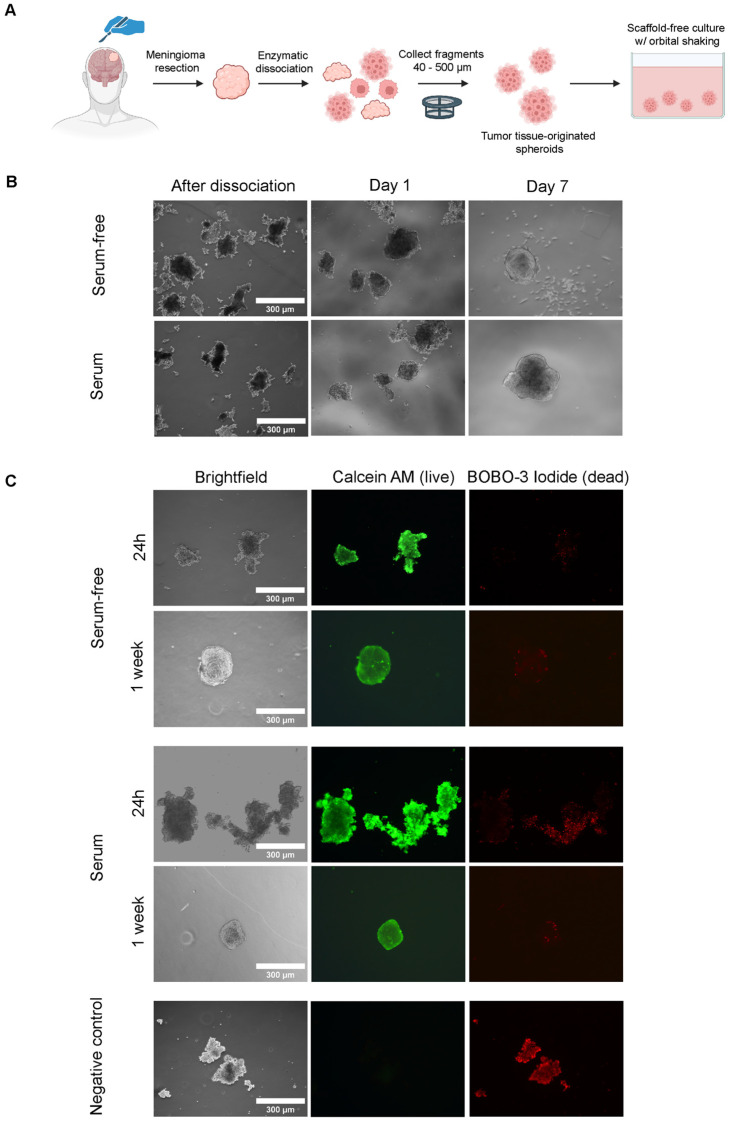
Generation of meningioma tumor tissue-originated spheroid model. Serum-free = growth factor-supplemented and serum = FCS-supplemented medium. (**A**) General overview of establishment of spheroid model from moment of resection to culturing. (**B**) Morphology of cultured meningioma fragments in different medium types directly after dissociation, at day 1 and day 7 of culturing. (**C**) Live/dead staining of meningioma fragments at day 1 and day 7 of culturing, as assessed by calcein AM (**middle** panel, green, live cells) and BOBO-3 Iodide (**right** panel, red, dead cells) staining. Images for a negative control (fragments treated with 70% ethanol), staining negative for Calcein AM and positive for BOBO-3-Iodide are shown in the **bottom** panel.

To establish spheroids with high yield from various starting amounts of meningioma tissue, we optimized a previously described enzymatic dissociation protocol [22], using Liberase TM, dispase, and DNAse I. Using this protocol, we were able to generate sufficient and highly viable meningioma fragments from diverse patient samples, independent of input tissue texture, within a 2 h time period from resection (see Figure 1A for workflow). Dissociation yielded fragments of heterogeneous sizes with rough edges that adapted a spheroidal morphology after overnight culture (Figure 1B). This spheroidal morphology further progressed during the first week of culture. Assessment of cell viability by live/dead staining indicated that some fragments contained dead cells and cellular debris after enzymatic dissociation, which were lost during culturing, yielding spheroids exhibiting high viability (Figure 1C). Cultures were performed in both growth factor-supplemented media and media supplemented with FCS. However, these medium types did not result in a visible difference in spheroid morphology and viability during the 7-day culture period (Figure 1C). Generation and culturing of spheroids was possible for all meningioma samples of different grades included in this study (Table 1).

### 3.2. Meningioma Spheroids Recapitulate Tissue Architecture and Meningioma Characteristics but Show Phenotypic Drift over Time

**Figure 2:**
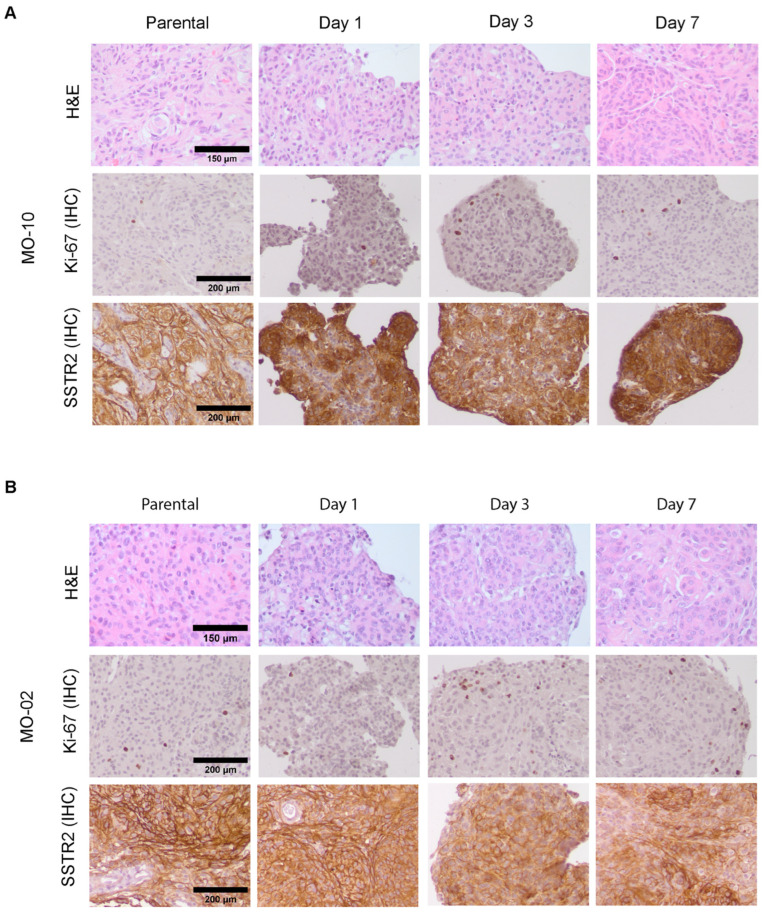
Assessment of spheroid phenotype by H&E and IHC for Ki-67 and SSTR2 compared to parental tumors. H&E staining (**top** panel) and IHC staining for Ki-67 (**middle** panel) and SSTR2 (**bottom** panel) for parental tumor tissue and spheroids cultured for 1, 3, or 7 days. Example images are shown for spheroids derived from MO-10 (**A**) and MO-02 (**B**), only for spheroids cultured in FCS-containing medium. For results for spheroids cultured in growth factor-supplemented medium, see Supplementary Figure S1.

To investigate if key tumor characteristics were retained during short-term spheroid culture (1 week), we first assessed tumor histological features by H&E staining. These stainings revealed that spheroids, e.g., those of MO-10 (Figure 2A), retained key histological features from their parental tumors, including whorls and nuclear inclusions. However, a subset of meningioma spheroids, e.g., those of MO-02 (Figure 2B), showed an increasingly aggressive phenotype compared to their parental counterpart over time during culturing, indicated by the presence of nucleoli, dark nuclei, and increased presence of mitotic figures, which for some meningiomas was already visible from day 1. 

Furthermore, IHC staining for Ki-67 as a proliferation marker showed that spheroids retained the low proliferative index typical for a WHO grade 1 meningioma (Figure 2A). From day 3 onwards, focal increases in Ki-67-positive cells, indicative of a more aggressive phenotype, could be detected, mostly in the spheroid periphery (Figure 2B).

As SSTR2, the target receptor for ^177^Lu-DOTA-TATE-based PRRT is considered a specific marker for meningiomas [23]; we additionally assessed SSTR2 levels in meningioma spheroids during culturing. Both the SSTR2 expression level and distribution (focal vs. diffuse) of parental tumors were maintained in spheroids (Figure 2A,B). However, starting from day 3 and progressing towards day 7, specific areas with a lower SSTR2 positivity could be identified in the majority of samples, corresponding to a decrease in SSTR2 levels during culturing. Again, the extent of SSTR2 loss was heterogeneous between patient samples and generally correlated with that of an increasingly aggressive phenotype over time as assessed by H&E and Ki-67 index. For example, SSTR2 was more retained in MO-10 (Figure 2A) than in MO-02 (Figure 2B) during culturing.

Together these data indicate that meningioma spheroids generally retain their key phenotypical features during short-term culturing, but that the culture method does induce progression of grade 1 meningiomas towards a more aggressive phenotype, together with gradual loss of SSTR2 expression. In addition, the extent of these changes varied between different patient samples. No clear differences in spheroid phenotype were observed between growth factor-supplemented and FCS-supplemented media types (Figure 2 and Figure S1).

### 3.3. ^177^Lu-DOTA-TATE Binds to Meningioma Spheroids via SSTR2

**Figure 3:**
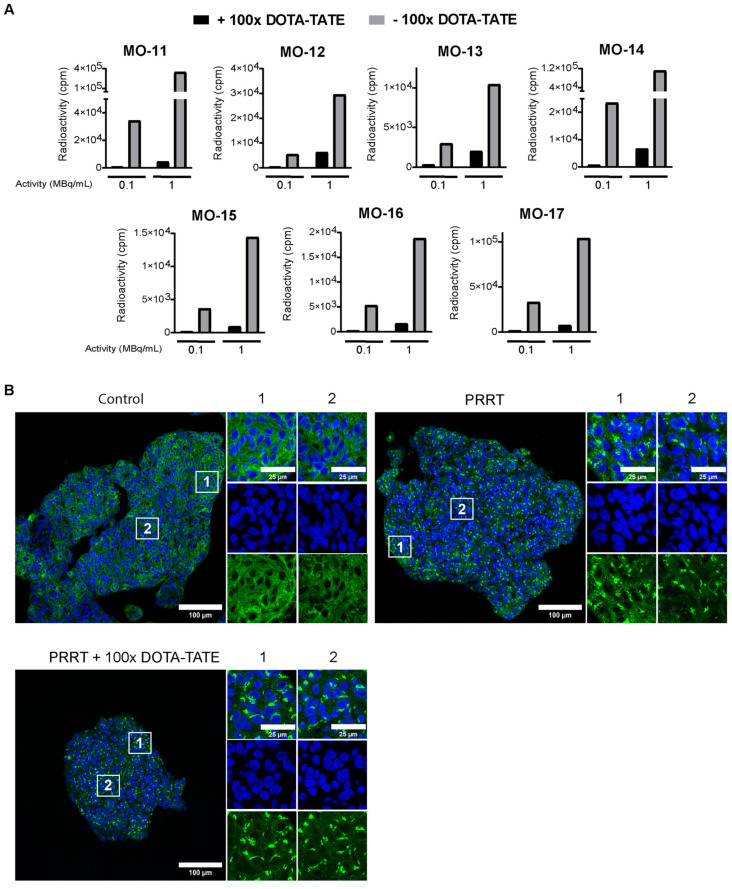
Binding of ^177^Lu-DOTA-TATE to meningioma spheroids. (**A**) Uptake assay determining quantity of radioactivity in counts per minute (cpm) in meningioma spheroids after ^177^Lu-DOTA-TATE treatment with (black bars) or without (grey bars) co-treatment with 100× molar excess of DOTA-TATE. For each meningioma, data are shown for spheroids incubated with 0.1 and 1 MBq/mL ^177^Lu-DOTA-TATE. (**B**) Representative tile scan overview from MO-10 of IF staining for SSTR2 (green) for control spheroids, spheroids treated with PRRT (1 MBq/mL) or spheroids treated with PRRT co-incubated with DOTA-TATE. DAPI (blue) was used as a nuclear counterstain. Directly after treatment with either PRRT or PRRT and DOTA-TATE, loss of membranous signal and intracellular accumulation of SSTR2 can be seen throughout the spheroid. White squares indicate the zoomed-in regions, shown right from each corresponding tile scan for the peripheral (1) or core (2) region of the spheroid.

Next, we examined whether we could use the described culture model to assess the short-term response to radiation therapies, focusing on PRRT. First, we investigated if meningioma spheroids could actively take up ^177^Lu-DOTA-TATE in vitro, using a panel of seven meningiomas (WHO grade 1 (n = 5); WHO grade 2 (n = 1); WHO grade 3 (n = 1)) (Table 1). Considering the observed phenotypical changes in vitro (Figure 2 and Figure S1), we limited the timepoint of the start of treatment to day 3 after the establishment of cultures. After incubation with 0.1 MBq/mL and 1 MBq/mL ^177^Lu-DOTA-TATE, spheroids of all meningioma samples showed a concentration-dependent uptake (Figure 3A). Importantly, spheroids that were co-incubated with 100× molar excess of unlabeled DOTA-TATE showed dramatically lower uptake than those only incubated with ^177^Lu-DOTA-TATE. This indicated that the measured radioactivity is specifically taken up via SSTR2. Unfortunately, due to variations in the input amount of tissue between tumors, a direct comparison in absolute uptake values between tumors was not possible.

To further characterize PRRT uptake, we stained spheroids for SSTR2 directly after treatment. While untreated spheroids showed clear membranous staining of SSTR2, spheroids treated with either PRRT alone or PRRT combined with DOTA-TATE displayed receptor internalization and intracellular accumulation (Figure 3B). Importantly, receptor internalization was visible over the entire spheroid diameter in a given section, confirming the complete penetration of ^177^Lu-DOTA-TATE into spheroids with sizes 40–500 µm, used for our culture method.

### 3.4. ^177^Lu-DOTA-TATE Induces DNA Damage in Meningioma Spheroids Correlating with SSTR2 Expression Levels

**Figure 4:**
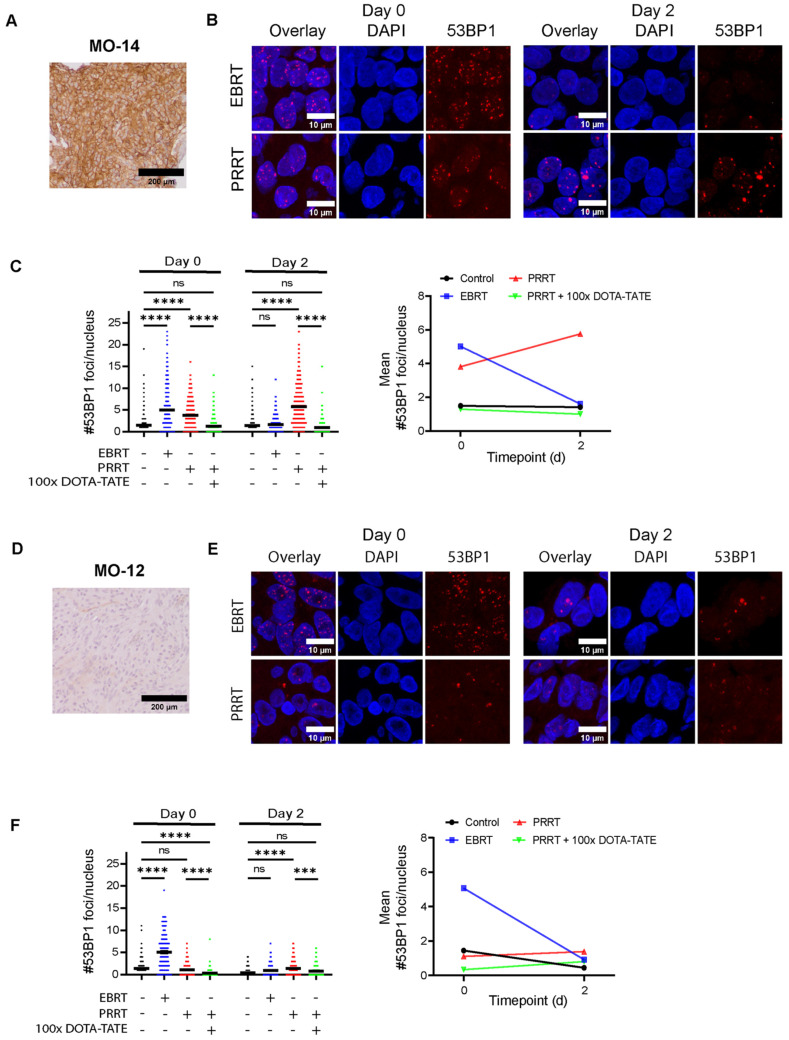
Assessment of DNA damage in meningioma spheroids after EBRT and PRRT. (**A**,**D**) IHC staining for SSTR2 in parental tumor tissue for a meningioma staining strongly (MO-14, (**A**) and weakly (MO-12 (**D**)) SSTR2-positive. (**B**,**E**) Representative images of 53BP1 IF staining (red) used for the quantifications in (**C**,**F**). Examples are shown for EBRT (2 Gy)- and PRRT (1 MBq/mL)-treated spheroids at both day 0 and day 2 post-therapy. DAPI (blue) was used as a nuclear counterstain. (**C**,**F**) Quantification of the number of 53BP1 foci per nucleus after EBRT, PRRT, or PRRT co-incubated with DOTA-TATE at day 0 or day 2 post-therapy for MO-14 (**C**) and MO-12 (**F**). Black horizontal bars indicate the mean. ns = not significant; *** *p* ≤ 0.001; **** *p* ≤ 0.0001. Line graphs on the right show the mean number of 53BP1 foci for each condition. Quantification for other meningioma samples can be found in Supplementary Figure S2.

After showing specific uptake of PRRT, we examined short-term therapy effects by assessment of DNA damage using 53BP1 foci as a double-strand break (DSB) repair marker [24]. In parallel, spheroids were treated with EBRT to compare directly to PRRT-induced DNA damage. As expected, EBRT initially induced an increase in 53BP1 foci numbers compared to controls in all meningioma samples (assessed 2 h post-EBRT) (Figure 4 and Figure S2). At day 2, 53BP1 foci numbers returned to control levels, indicating the repair of most DSBs. For 3 out of 7 meningiomas (MO-10, MO-13, and MO-15), a significantly higher number of 53BP1 foci was detected compared to the control at day 2 post-EBRT, indicating incomplete repair of IR-induced DSBs.

In contrast, while PRRT also showed an increase in 53BP1 foci numbers at day 0 (assessed directly post-PRRT), meningioma spheroids still showed significantly elevated 53BP1 levels at day 2 post-PRRT, correlating with the extended irradiation time of PRRT as compared to EBRT (Figure 4 and Figure S2). Co-incubation with 100× excess DOTA-TATE significantly attenuated the induction of DNA damage, leading to lower foci numbers as compared to spheroids treated with only PRRT at both day 0 and day 2 post-therapy. This showed that DNA damage after PRRT was specifically induced through binding to SSTR2. This observed pattern of DNA damage induction by PRRT could be detected for six meningioma samples staining strongly positive for SSTR2 (Figure 4A–C and Figure S2). Interestingly, we could not detect a significant increase in 53BP1 foci numbers compared to controls at both day 0 and day 2 post-therapy for one meningioma sample (MO-12), for which tumor tissue stained only weakly for SSTR2 (Figure 4D–F). These observations confirmed that meningioma spheroids could be used to assess the short-term response to radiation therapies including PRRT.

## 4. Discussion

PRRT has been proposed as a new radiation treatment approach for meningiomas with high surgical morbidity or recurrent meningioma [25]. While currently available clinical data points towards promising results on efficacy and toxicity [9], further research is necessary to elucidate the full potential of radionuclide therapies in these tumors, both from a preclinical and clinical perspective. For this, the availability of relevant meningioma models is essential. Here, we developed a patient-derived 3D meningioma culture model that can be used to assess the response to EBRT and PRRT.

Our model employs tumor tissue-originated spheroids in a scaffold-free culture method, which is easy to use and can be established quickly after resection. This method is based on partial dissociation of tumor tissue and subsequent culturing of tumor cell clusters. In contrast to patient-derived tumor culture approaches where tissue is completely dissociated to single cells and then reaggregated into 3D structures, such as recently described for meningiomas [26], our method retains the original tumor architecture and (partial) cell–cell contacts. This culture method, sometimes referred to as cancer tissue-originated spheroids (CTOS) has been successfully used for a variety of tumor types, including colorectal, urothelial, and bladder cancer [27,28,29]. Previously, we have explored the use of alternative culture approaches such as organotypic tumor slices. These slices have been shown to faithfully mimic various types of tumors, including their microenvironment, which would pose significant advantages in the preclinical assessment of therapeutics [30,31,32,33]. Unfortunately, due to the highly variable texture of meningioma tissue, this method could not be utilized in a consistent manner in our hands. 

The use of patient-derived 3D culture models such as tumor tissue-originated spheroids is highly relevant in the context of radiobiology. Cells have consistently been shown to exhibit higher radioresistance in a 3D environment as compared to 2D [34], for example through differences in DNA damage induction and repair [35,36]. In addition, for targeted radionuclide therapy approaches such as PRRT, dosimetric parameters such as the dose distribution will be different between 2D and 3D culture settings [37]. For example, an increased contribution of cross-dose between cells can be expected in 3D. Finally, using a patient-derived model for radiobiological research allows for investigating the patient-specific characteristics that contribute to the radiation response, which is heavily understudied in meningiomas [6]. 

In this study, meningioma spheroids could be generated with high yield and within a short timeframe, and they retained viability and key histological tumor characteristics during short-term culturing. Despite these promising results, for a subset of meningioma samples, we detected phenotypical changes in cultured spheroids, starting relatively early after dissociation. These changes included histological features associated with more aggressive tumor behavior, including nucleoli, and an increase in mitotic figures, mostly in the spheroid periphery. As the spheroid periphery is exposed to the highest level of culture stress, including the highest concentration of culture medium components (such as growth factors) and fluid shear stress during shaking, it is not surprising that the observed phenotypical changes were most evident in this region. In addition, we detected a decrease in SSTR2 expression over time for meningioma spheroids. Rapid decreases in the expression of SSTR2 and other hormone receptors such as progesterone receptor (PR) during culturing of primary meningioma cells have been reported before [26,38,39]. This might be caused by dedifferentiation of cells when dissociated from their native tumor environment. The fact that the extent of these phenotypical changes was heterogeneous between tumors, ranging from almost none to highly evident after short culturing periods, suggests that the success of the used culture method might be dependent on patient-specific traits, such as genetic background. In addition, considering the loss of the benign phenotype of some grade 1 meningiomas during culturing, our developed culture method might be more suitable for meningiomas of higher grades. These hypotheses should be confirmed using larger meningioma cohorts including more WHO grade 2 and 3 tumors. For the development of the culture method, we only used WHO grade 1 meningiomas, which can be considered a limitation of this study, while for therapy assessment studies we also used 1 WHO grade 2 and 1 WHO grade 3 meningioma (Table 1).

To enable longer-term culturing of meningiomas of all WHO grades, subsequent experiments need to focus on optimizing critical components for this culture system. Culturing of low-grade meningiomas might require additional supporting factors as compared to the scaffold-free system used here. These include specific medium components, further reduction in culture stress, and incorporation of a culture scaffold such as a basement membrane extract. For example, Yamazaki et al. recently described the establishment of an organoid system for intracranial meningiomas [19], based on culturing in basement membrane extract. Importantly, the authors showed that organoids retained features of their parental tumors for several weeks, including SSTR2 expression and proliferation index. 

Despite the need for further optimization of the spheroid model, we were able to validate its use to assess the short-term response to radiation therapies such as EBRT and PRRT, using 53BP1 foci levels as a readout. The visualization of therapy-induced DSBs, e.g., by staining for γ-H2AX or 53BP1 foci, has increasingly been used in patient-derived culture models to assess the IR-response [40,41,42]. For PRRT, we showed a prolonged presence of elevated foci levels as compared to EBRT, which was alleviated by co-incubation with 100× molar excess DOTA-TATE, demonstrating specific receptor binding and subsequent damage induction during, at least, multiple days. Here, we measured 53BP1 foci at an early (2 h post-EBRT and directly post-PRRT; day 0) and late (day 2 post-EBRT and -PRRT) timepoint. The level of DSB repair foci could also be used as a readout to assess the efficacy of PRRT at various other timepoints. 

The selection of additional readouts relevant to the response to EBRT and PRRT, both short- and long-term, remains to be determined. Cell death in the form of apoptosis, sometimes combined with assessment of proliferation, is used to assess the short-term response to radio- or chemotherapy in patient-derived tumor models [43,44]. To what extent apoptosis is a relevant endpoint after irradiation of meningiomas is currently unclear. In addition, due to the intrinsically low proliferative index of the majority of meningiomas, assessment of a reduction in proliferation markers is not a suitable readout here. Importantly, for the evaluation of the radiobiological effects of PRRT, long-term effects are highly relevant. This is partly due to the fact that PRRT exhibits a low dose rate and the half-life of lutetium-177 is 6.7 days [45], leading to a protracted exposure time to IR. For example, recent data suggest that senescence is a highly relevant long-term outcome after PRRT in vivo, where the delayed induction of senescence occurred in tumor regions with the retainment of lutetium-177, detected up to 42 days post-treatment [46]. 

As current knowledge on meningioma radiobiology is limited, the critical determinants of the meningioma radiation response remain to be determined. In addition to the WHO grading system for meningiomas [47], various tumor classification systems have been proposed to improve meningioma risk stratification and (radio)therapy response prediction, including RNA sequencing [48], DNA methylation profiling [49], and integrated risk score based on a combination of histology, epigenetics, and copy-number variations [50]. Recently, a large study discovered and validated a 34-gene expression risk scoring panel predicting the response to postoperative radiotherapy [51]. Functional assessments of the IR-induced DNA damage response, such as the readout of 53BP1 foci we have used here, not only provide a valuable tool to identify more critical factors for the radiation response of meningiomas, but in the future might also be used to directly predict the response of individual patients to IR-based therapies. Interestingly, for a subset of the meningiomas in our cohort, we detected a significantly elevated level of 53BP1 foci in spheroids at day 2 post-EBRT, while for other samples, foci levels returned completely to control levels at this timepoint. To further characterize this observation, future research should focus on a larger cohort of meningiomas, potentially directly coupling the response to relevant molecular tumor characteristics identified in other studies and to clinical outcomes, ultimately verifying the predictive power of patient-derived meningioma models.

## 5. Conclusions

In conclusion, we have demonstrated the proof-of-concept for an easy-to-use patient-derived meningioma 3D culture model, retaining key features of the parental tumors, that can be employed to assess the short-term radiobiological response to EBRT and PRRT. Further optimization of culture conditions should enhance the degree of tumor mimicry, including therapy response, and pave the way to the assessment of long-term radiobiological responses to radionuclide therapies such as PRRT. In addition, the model might be employed to understand and test the response to other relevant candidate therapies for meningiomas.

## Figures and Tables

**Table 1:** Collected meningioma samples for optimization of culture conditions and therapy assessment, including corresponding patient characteristics. None of the patients had received irradiation treatment prior to these experiments.

Tumor Code	Grade	Tumor Location	Experimental Use	Patient Info (Sex, Age)
MO-1	1	Right parietal	Culture conditions	F, 56
MO-2	1	Left frontal, parasagittal	Culture conditions	F, 70
MO-3	1	Left frontal parasagittal	Culture conditions	F, 63
MO-4	1	Right frontal	Culture conditions	F, 61
MO-5	1	Right parasagittal	Culture conditions	F, 51
MO-6	1	Skull base	Culture conditions	F, 74
MO-7	1	Right frontal	Culture conditions	M, 57
MO-8	1	Tuberculum sellae	Culture conditions	M, 71
MO-9	1	Cerebellar	Culture conditions	F, 54
MO-10	1	Right frontal	Culture conditions + Therapy	F, 55
MO-11	2	Skull base	Therapy	M, 60
MO-12	1	Left occipital	Therapy	M, 54
MO-13	1	Right anterior clinoid process	Therapy	M, 72
MO-14	3	Right frontotemporal	Therapy	F, 57
MO-15	1	Falx	Therapy	F, 42
MO-16	1	Right tentorial	Therapy	M, 30

## Data Availability

Data generated and analyzed during this study are available from the corresponding author upon reasonable request.

## Supplementary Materials

The following supporting information can be downloaded at: https://www.mdpi.com/article/10.3390/cancers16142515/s1, Figure S1: Histological and immunohistochemical assessment of spheroid phenotype, cultured in growth factor-supplemented medium. H&E-staining (top panel) and IHC staining for Ki-67 (middle panel) and SSTR2 (bottom panel) for spheroids cultured for 1, 3 or 7 days. Example images are shown for spheroids derived from MO-10 (A) and MO-02 (B); Figure S2: Assessment of DNA damage meningioma spheroids after EBRT and PRRT. Quantification of the number of 53BP1 foci per nucleus after EBRT (2 Gy), PRRT (1 MBq/mL) or PRRT co-incubated with 100× excess DOTA-TATE at day 0 or day 2 post-therapy. All meningiomas not included in Figure 4 are shown. For each meningioma, IHC staining for SSTR2 is shown on the left. Black horizontal bars represent the mean. ns = not significant; * *p* ≤ 0.05; ** *p* ≤ 0.01; **** *p* ≤ 0.0001.
