# Supplementary material for: Investigating the Radiobiological Response to Peptide Receptor Radionuclide Therapy Using Patient-Derived Meningioma Spheroids
Source: Cancers (Basel). 2024 Jul 11;16(14):2515. doi: 10.3390/cancers16142515 (PMC11275064; doi:10.3390/cancers16142515)
Supplement: Supplementary file 1 [file cancers-16-02515-s001.zip › cancers-3075904-supplementary.pdf]

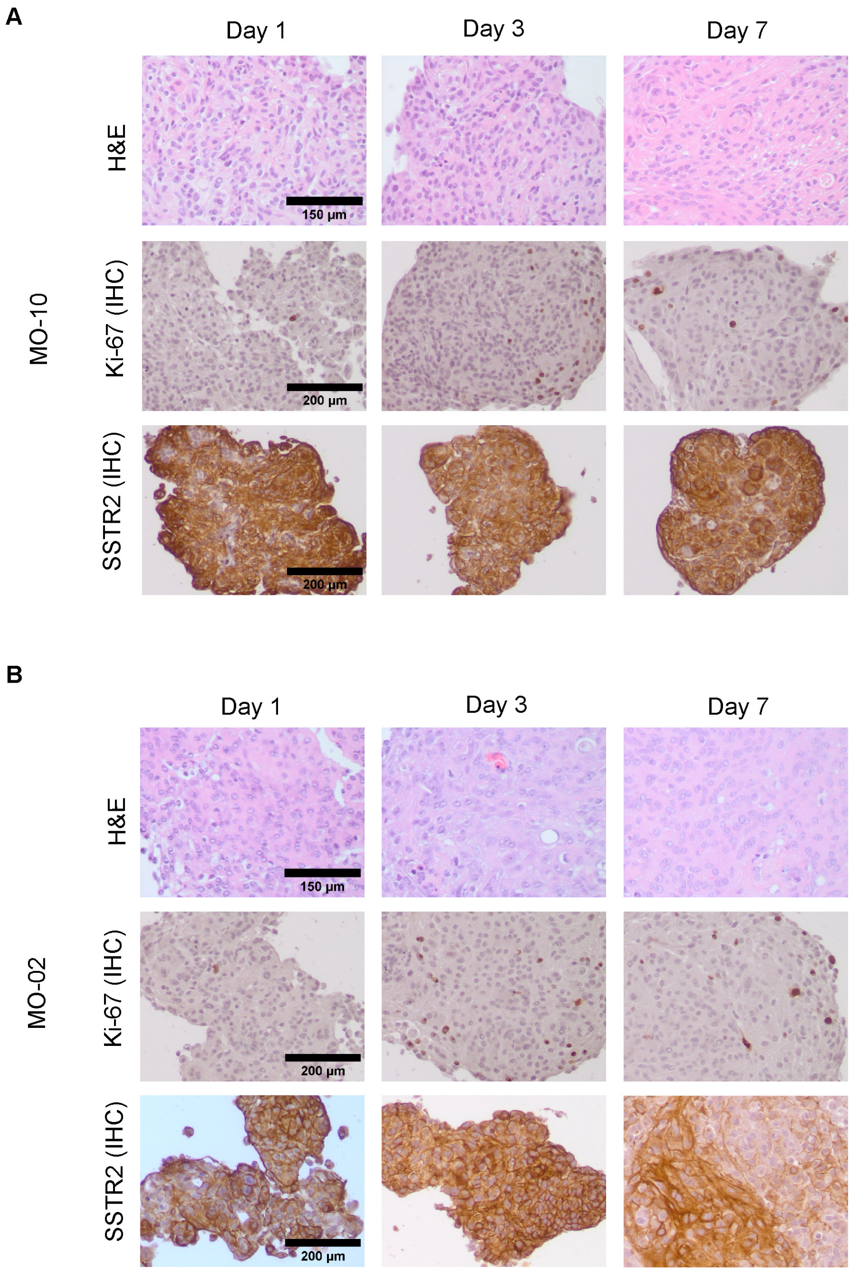

Figure S1. Histological and immunohistochemical assessment of spheroid phenotype, cultured in growth factor-supplemented medium. H&E-staining (top panel) and IHC staining for Ki-67 (middle panel) and SSTR2 (bottom panel) for spheroids cultured for 1, 3 or 7 days. Example images are shown for spheroids derived from MO-10 (A) and MO-02 (B).

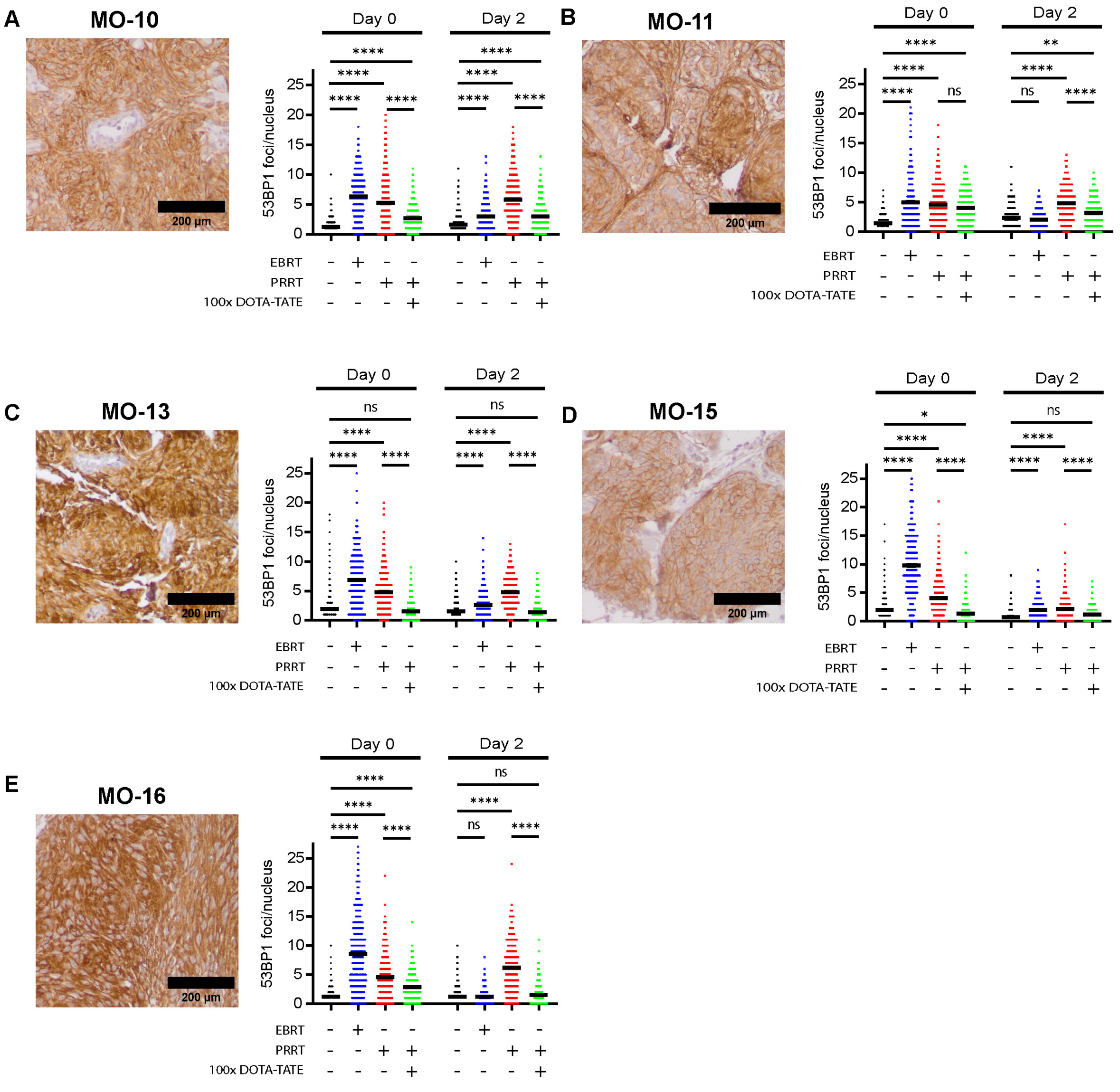

Figure S2. Assessment of DNA damage meningioma spheroids after EBRT and PRRT. Quantification of the number of 53BP1 foci per nucleus after EBRT (2 Gy), PRRT (1 MBq/mL) or PRRT co-incubated with 100× excess DOTA-TATE at day 0 or day 2 post-therapy. All meningiomas not included in Figure 4 are shown. For each meningioma, IHC staining for SSTR2 is shown on the left. Black horizontal bars represent the mean. ns = not significant; * $p \leq 0.05$; ** $p \leq 0.01$; **** $p \leq 0.0001$.
